# Can Functional Cognitive Assessments for Children/Adolescents Be Transformed into Digital Platforms? A Conceptual Review

**DOI:** 10.3390/children12101384

**Published:** 2025-10-14

**Authors:** Yael Fogel, Naomi Josman, Ortal Cohen Elimelech, Sharon Zlotnik

**Affiliations:** 1 Department of Occupational Therapy, Ariel University, Ariel 4070000, Israel; 2 Department of Occupational Therapy, Faculty of Social Welfare and Health Sciences, University of Haifa, Haifa 3498838, Israel; njosman@univ.haifa.ac.il (N.J.); ocoheneli@staff.haifa.ac.il (O.C.E.); 3 Department of Occupational Therapy, Zefat Academic College, Jerusalem St 11, Safed 1320611, Israel; sharon.zl@zefat.ac.il (S.Z.)

**Keywords:** performance-based assessment, ecological validity, executive functions, cognitive assessment

## Abstract

**Highlights:**

**What are the main findings?**

- Most functional cognitive assessments (n = 13) for children and adolescents with high ecological validity remain unavailable in digital formats, highlighting a significant gap between traditional tools and technology-based solutions.
- Digitization of these assessments offers potential benefits, such as improved accessibility, precision in data collection, and scalability; however, replicating real-life contexts and capturing strategy use digitally remains challenging.

**What is the implication of the main finding?**

- Innovative digital tools are needed that successfully achieve high ecological validity by preserving real-world context and observation of strategies, while maximizing digital availability through advanced technologies (e.g., VR, mobile platforms).
- The proposed conceptual framework can guide clinicians, researchers, and developers in prioritizing features for future technology-enhanced assessments, promoting evidence-based, context-sensitive evaluation practices.

**Abstract:**

Background/Objectives: Functional cognition, integrating cognitive abilities during real-life task performance, is essential for understanding daily functioning in children and adolescents. Traditional paper-based cognitive assessments in controlled environments often lack ecological validity. Although performance-based assessments more accurately represent functioning in natural contexts, most have not been transformed into digital formats. With technology increasingly embedded in education and healthcare, examining the extent/nature of adaptations, benefits, and challenges of digitizing these tools is important. This conceptual review aimed to (1) examine the extent/nature of traditional performance-based cognitive assessments adapted into digital platforms, (2) compare ecological validity/scoring metrics of traditional and digital tools, and (3) identify opportunities and propose recommendations for future development. Methods: We used an AI-based tool (Elicit Pro, Elicit Plus 2024) to conduct a literature search for publications from the past decade, focusing on transformations of traditional assessments into digital platforms for children and adolescents. This initial search yielded 240 items. After screening, 45 were retained for manual review. Studies were extracted based on their discussion of the assessments (traditional or digital) and assessment tools used. Ultimately, 13 papers that met the inclusion criteria were evaluated based on units of analysis. Results: The analysis yielded three units. The first unit focused on digital transformation trends: four assessments (31%) were converted to digital platforms, two (15%) were developed as native digital tools, and the majority (seven, 54%) remained traditional. In the second unit, assessments were evaluated according to ecological validity and digital availability, demonstrating that assessments with high ecological validity tended not to be digitally accessible. The third unit synthesized scoring metrics, identifying eight distinct cognitive domains. Conclusions: Digitizing functional cognitive assessments offers greater accessibility, precision, and scalability, but replicating real-world contexts remains challenging. Emerging technologies may enhance ecological validity and support development of effective, technology-enhanced assessment practices.

## 1. Introduction

Cognitive abilities play a pivotal role in performing daily activities, from simple routines to complex tasks that require executive and metacognitive skills. For individuals with cognitive impairments, even basic activities of daily living (ADLs) may present significant challenges [1,2]. In occupational therapy and related disciplines, the concept of *functional cognition*, the integration of cognitive abilities while performing real-life tasks, has become increasingly relevant [3,4].

Although there is no universally accepted definition of *functional cognition*, it is generally conceptualized as the dynamic interplay between cognitive processes (e.g., attention, memory, executive functions [EFs]), performance skills (e.g., motor abilities), and environmental and task demands [5,6,7]. Functional cognition is best evaluated through direct task performance rather than isolated cognitive testing because direct tasks more accurately reflect an individual’s capacity to manage everyday demands [8,9,10].

Assessing functional cognition is a cornerstone of understanding daily performance, particularly in children and adolescents, whose participation in ADLs and instrumental ADLs is still developing [6,7,11]. Unlike traditional cognitive tests that evaluate isolated domains, functional cognition assessments emphasize the integration of cognitive abilities, such as attention, memory, and EFs, with motor skills, environmental demands, and task complexity [5,6,7]. As such, they provide a more ecologically valid picture of the child’s or adolescent’s ability to function in real-life contexts.

*Ecological validity* describes how accurately an assessment predicts performance in natural environments [12]. It includes two essential components: representativeness and generalizability. *Representativeness* describes how closely the assessment reflects real-life situations, rather than artificially controlled tasks in a laboratory. *Generalizability* is the degree to which the performance in the assessment predicts functioning in everyday settings [3,4]. In this sense, performance-based assessments that simulate or observe actual daily activities offer significant advantages over traditional formats, particularly in capturing the real-world impact of cognitive challenges.

Thus, functional cognition is best evaluated in the context of actual task performance, that is, *occupational performance*, rather than through decontextualized or standardized conditions [8,9]. Performance-based approaches, including direct observation in naturalistic environments or the use of simulated tasks, provide more accurate insight into the interaction between cognitive processes, motor abilities, and environmental demands [13].

Moreover, such context-sensitive assessments align with professional standards and national expectations for demonstrating meaningful outcomes in clinical practice [14]. They also support identifying concrete intervention targets in domains such as schoolwork, play, social participation, and self-care, where cognitive demands intersect with emotional, behavioral, and contextual factors [9].

Traditional assessments of functional cognition and EFs in children and adolescents have relied predominantly on standardized tests and proxy-report questionnaires, typically using paper-and-pencil formats. These assessments are generally administered in structured, controlled environments like clinics or schools [15]. A common example is the Behavior Rating Inventory of Executive Function, widely used to assess EF-related behaviors in home and school contexts through parents’ and teachers’ reports [16]. Another widely applied tool is the Delis–Kaplan Executive Function System, which evaluates a range of verbal and nonverbal EFs in children and adults through standardized tasks [17]. Although these traditional assessments are established and effective for identifying cognitive deficits, they do not fully reflect how children and adolescents function in their natural, everyday environments, thus limiting the assessments’ ecological validity.

In contrast, performance-based assessments focus on real-world tasks and appear to offer greater ecological validity. These tools aim to provide a more in-depth understanding of an individual’s functional cognitive abilities by capturing their use of strategies, performance patterns, habits, routines, and contextual and environmental supports rather than evaluating isolated cognitive domains such as attention, memory, or EFs [14,18]. Because these assessments simulate or directly observe performance in meaningful daily activities, they are seen as more representative of actual functioning and recognized as valuable alternatives to traditional approaches [3,4]. Performance-based assessments are particularly beneficial when conducted in real-life or simulated contexts that account for the dynamic interplay between cognitive processes, motor skills, and environmental demands [13].

Bridging these two approaches, the Cognitive Functional Evaluation-Extended model [19,20] integrates traditional cognitive tests with performance-based measures to provide a comprehensive and context-sensitive evaluation of functional cognitive in children and adolescents. This model combines the assessment of cognitive abilities, daily performance, and environmental influences to reflect the dynamic and interactive nature of cognitive functioning. It enables clinicians to develop a holistic understanding of a child’s cognitive capabilities and how these abilities manifest in everyday life, supporting more targeted and ecologically valid interventions.

The environments in which individuals function today—including schools, healthcare centers, workplaces, and social settings—are increasingly embedded with technology [21,22]. Considering these rapid technological developments, growing attention has been directed toward creating instruments that use digital platforms to assess functional cognition. Over the past decade, platforms such as virtual reality (VR) environments, wearable sensors, desktop-based software, and mobile applications have been shown to enhance the ecological validity of cognitive assessments by facilitating real-time evaluation in more naturalistic contexts [23].

Technology-based assessments offer a range of benefits. They provide increased accessibility, allow the simulation of diverse and complex real-life scenarios, and support data collection that is more precise and objective through automated scoring and advanced analytics [24]. In addition, the expansion of telerehabilitation and remote healthcare services has opened new avenues for evaluating cognitive functioning outside of traditional clinical settings, making assessments more flexible and scalable [25,26].

Despite their promise, technology-based assessment tools pose several important challenges that must be addressed. These include the need for rigorous methodological precautions to ensure validity, reliability, and consistency in measuring outcomes [11,25,27]. Furthermore, for these tools to justify the resources required for their development and implementation, they must not only replicate the capabilities of traditional assessments but also provide added value by improving measurement sensitivity, relevance, or efficiency [28].

Although research on the feasibility of converting existing cognitive assessment tools into technology-based platforms is ongoing, most studies have focused on general cognitive tests. These efforts have explored whether digital platforms can maintain or enhance the accuracy of assessment outcomes and the potential benefits and limitations of such conversions compared to traditional methods [29]. However, to our knowledge, there has been no specific investigation into the transformation of performance-based assessments of functional cognition into digital tools. Hence, there is a significant gap in the current literature, particularly given the recognized ecological value of performance-based approaches and the increasing integration of technology into educational and healthcare environments.

**Figure 1:**
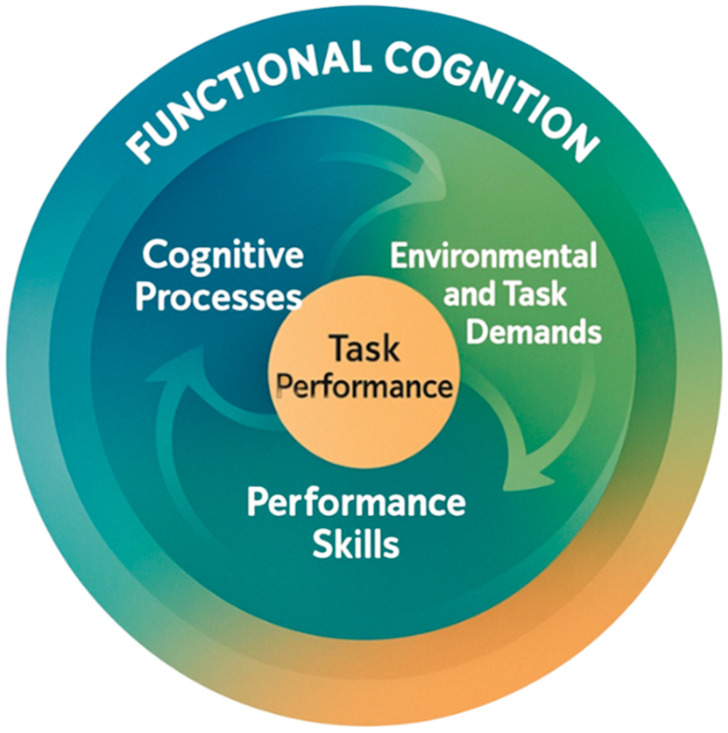
The components and interrelations of functional cognition.

Against this backdrop, our conceptual review explores the feasibility, effectiveness, and potential advantages of transforming traditional performance-based assessments of functional cognition into digital platforms, focusing specifically on assessments for children and adolescents. Figure 1 illustrates the components of functional cognition and their interrelations. The figure leans on the established definition of functional cognition to better describe this complex term.

Building on the current literature and theoretical perspectives, this review aims to contribute to the evolving discussion on how technological innovations can support more context-sensitive and developmentally appropriate cognitive assessments. By highlighting emerging directions and identifying key considerations for future development, the review aspires to advance more accurate, effective, and ecologically valid assessment tools that can be integrated into real-world clinical and educational settings. Hence, the specific objectives of this conceptual review are to

1. Examine the current extent and nature of adaptations of traditional performance-based cognitive assessments into digital platforms.
2. Compare the ecological validity and scoring metrics of traditional tools versus digital platforms.
3. Identify opportunities and propose evidence-informed recommendations for the future development of digital platforms for functional cognition assessment in children and adolescents.

## 2. Materials and Methods

This study follows Jaakkola’s [30] template for constructing a conceptual review. In this method, the researchers classify and organize concepts into categories based on shared characteristics to facilitate clearer understanding and analysis. To gather and perform an initial search and analysis of relevant literature for this review, we utilized Elicit [https://elicit.com/] using the Pro license. Elicit is an AI-powered research assistant designed to facilitate evidence synthesis by retrieving, summarizing, and structuring information from academic sources. Elicit searches across over 126 million academic papers from a corpus of open-access and non-open-access studies across all academic disciplines [https://support.elicit.com/en/categories/146369, accessed on 29 September 2025]. The study selection process was performed by three independent researchers. An AI-assisted search, completed in September 2025, identified an initial 240 items. After a title and abstract screening, 45 were retained for manual review. Following a full-text analysis, 13 studies met the final inclusion criteria. The implementation of artificial intelligence tools adheres to the TITAN guidelines (for details, see Supplementary Materials).

The data extraction procedure involved the following steps:

1. Structured search query: The query was entered into the Elicit “Find Papers” feature, which retrieves relevant academic articles based on the prompt, “Find papers related to the following topic—transforming traditional functional cognitive assessments into digital platforms: feasibility, effectiveness, and potential advantages for children and youth.”
2. Filtering and refining results: using built-in filters for publication year (e.g., last 10 years to focus on recent advancements), as well as English-language publication
3. Extracting key information in the following categories: (a) title, authors, and publication year, link to full text, and number of its citations; (b) abstract summary; (c) performance-based (yes/no); (d) transformation to digital platform (yes/no); (e) remote administration (yes/no); and (f) advantages and disadvantages.
4. Based on this selection, another thorough manual process was applied. This second analysis focused on identifying manuscripts that discussed performance-based cognitive assessments on either traditional or digital platforms. Of the initial 240 papers, the resulting data totaled 45 papers, which were exported into a spreadsheet for further in-depth manual analysis performed by all the researchers.
5. To mitigate potential biases from the AI-assisted search, such as the under- or over-retrieval of certain literature, a thorough manual review of all results was conducted. This manual screening involved excluding papers that discussed only intervention tools (e.g., computer programs, mobile applications, web and video conferencing platforms, computerized cognitive behavioral therapy interventions, cognitive training, and neuro-feedback) and not assessment tools. Non-performance-based cognitive assessments, such as interviews, were also excluded. Papers were also excluded if they involved participants other than children or adolescents or the researchers had not identified the selection as an academic manuscript. Thirteen papers covering various assessment methods, including traditional paper-and-pencil tests, tablet-based assessments, computerized neuropsychological tests, game-based assessments, and remote teleassessments, remained.

From the 13 articles that met the inclusion criteria, one unique assessment tool was identified and analyzed from each. Therefore, this study is based on the analysis of 13 assessment tools, with each tool counted only once. This analysis process also involved multiple stages: (1) Screening: Abstracts were reviewed to ensure relevance. (2) Full-text review: Articles meeting the criteria were reviewed in detail. (3) Discussions about performance-based assessments were further analyzed for the advantages or disadvantages of transformation. (4) Data extraction: We identified key concepts to establish the units of analysis. These key concepts were structured into tables and categorized, as detailed in the Results.

## 3. Results

Three key units of analysis emerged in our analysis of data from all 13 assessments regarding challenges and implications of digital cognitive assessment platforms: (a) digital transformation trends, (b) ecological validity and digital platforms, and (c) scoring metrics across cognitive domains.

### 3.1. Trends in Adapting Traditional Performance-Based Cognitive Assessments into Digital Platforms

Table 1 presents an overview of 13 cognitive performance-based assessment tools targeting various populations of children and adolescents, assessment methodologies, and their digital-platform transformations. Among those assessments, four (31%)—the Children’s Memory Test (CMT), Tower of London, Weekly Calendar Planning Activity (WCPA), and Wisconsin Card Sorting Test for children (WCST)—had been converted to digital platforms. In addition, two (15%) assessments—the Adaptive Cognitive Evaluation Explorer (ACE-X) and Cambridge Neuropsychological Test Automated Battery (CANTAB)—had been digitized natively. The other seven (54%), particularly EF and general cognitive assessment tools, remained entirely traditional.

The 13 assessments addressed diverse populations, focusing on general cognitive abilities, attention deficit hyperactivity disorder, autism spectrum disorders, EF deficits, learning disabilities, and neurodevelopmental delays. Newly added tools, such as the WCPA [31] and the Children’s Cooking Task (CCT) [32], provided ecologically valid measures of functional cognition, emphasizing real-world EF challenges.

**Table 1:** An overview of 13 cognitive performance-based assessments.

Assessment	Purpose: To …	Description	Population: Developed for …	Scoring Metric	Age Range (Years)	Psychometric Properties: Reliability and Validity Types Assessed	Digital Platform
**Weekly Calendar Planning Activity (WCPA)** [33]	examine impact of EFs difficulties on the ability to perform daily activities involving multiple steps	Participants enter a list of appointments on a weekly schedule according to specified rules. There are three difficulty levels.	adults and adapted for children and adolescents	Number of accurate meetings, rules followed, number of strategies, planning time, total time, efficiency score	6–21	Interrater reliability [34], test–retest reliabilities among college student [35], discriminate validity [31,35,36]. There is normative data for adolescents 12–18 years [37].	No digital version available
**Test of Everyday Attention for Children (TEA-Ch)** [38]	measure multiple aspects of attention. The second edition (TEA–Ch2) [39] provides a simplified arrangement for ages 5 to 7 years and an extended arrangement for ages 8 to 15 years.	A battery of game-like assessments comprising nine distinct tasks	adults and adapted for children and adolescents	Sustained attention, selective attention, and attentional control	6–16	Test–retest reliability [40], convergent validity [40], discriminate validity [41,42], construct validity [41,42,43]	Computer program measures reaction times, accuracy, and scores as part of TEA-Ch2
**Behavioral Assessment of the Dysexecutive Syndrome for Children (BADS-C)** [44]	evaluate EF through tasks that simulate real-life scenarios and problem-solving demands.	A battery of tasks, including the Playing Cards, Water, and Key Search tests and three versions of the Zoo Map Test.	adults and adapted for children and adolescents	Total time, planning time, and number of errors	8–16	Interrater reliability [44], ecological validity, construct validity [45], construct validity (e.g., [46]), discriminate validity (e.g., [47], concurrent validity [47]. Norms are available from 7 years old [44].	No digital version available
**Children’s Cooking Task** [48]	examine EF, problem-solving, and sequencing skills through a cooking task simulation.	Participate in preparing a chocolate cake and juice using the recipe provided.	adults and adapted for children and adolescents	Goal accomplishment, dangerous behavior, need for adult assistance, total time, total number of errors additions, omissions, comments/ questions, estimation errors, substitution sequence errors, control errors, context neglect, environmental adherence, purposeless actions and displacements, dependency, inappropriate behavior	8–20	Internal consistency, test–retest reliability, discriminant validity, concurrent validity [32,48]	No digital version available
**Do-Eat performance-based assessment** [49]	evaluate areas of strength and difficulty in activities of daily living and instrumental activities of daily living among children with various disorders and help define therapeutic goals for occupational therapy intervention focusing on motor, EF, sensory, and emotional skills	Conducted in a natural setting, involving three tasks: make a sandwich, prepare chocolate milk, and complete a certificate of achievement.	children with neurodevelopmental disorders	Total time, total score, cue scores, sensory motor skills, EF skills (attention, initiation, sequencing, shifting, spatial organization, temporal organization, inhibition, problem-solving, remembering instructions), and task performance.	5–8	internal consistency, interrater reliability, construct validity, concurrent validity [49,50]	No digital version available
**Children’s Kitchen Task assessment** [51]	assess EFs and process skills during cooking activities, focusing on problem-solving and error detection.	A Play-Do task accompanied by written and pictorial instructions. The child receives examiner-provided cues as needed to successfully complete the activity.	adults and adapted for children and adolescents	Total time, total score, number of cues, organization score	8–12	Interrater reliability, internal consistency [51,52]	No digital version available
**Preschool Executive Task Assessment** [53]	assess EFs among young children and determine the level of assistance they need to accomplish the task	The child is instructed to draw a caterpillar picture. The child receives a box containing the necessary equipment and a comprehensive illustrated instruction book.	preschool children	Total time, total cues, total score, performance measure (working memory, organization, emotional ability, distractibility)	3–6	Interrater reliability [53], concurrent validity [52,54]	No digital version available
**Children’s Memory Test (CMT)** [55]	measure aspects of memory like immediate and delayed recall and meta-memory abilities in children (version 2, CMT-2, is available)	Memory task that involves four scenes relating to everyday living situations, each containing 20 pictures of objects.	adults and adapted for children and adolescents	Immediate recall, delayed recall, meta-memory (performance, prediction, performance estimation), and strategy used	5–16	Internal consistency, content validity, construct validity, concurrent validity [56]	Transferred to digital format
**Cambridge Neuropsychological Test Automated Battery (CANTAB)** [57]	assess cognitive abilities, such as visual memory, visual attention, and working memory/planning	Computerized neuropsychological tests that assess various cognitive functions. In this flexible battery, the researcher can select a subtask based on the participant’s interests.	adults and adapted for children and adolescents	Attention and psychomotor speed (mean reaction time, correct responses, false alarms, omission errors, sensitivity index, response variability, movement time, EF (number of problems solved, total errors, mean initial thinking time between/within errors, total stages completed, pre-extradimensional shift errors, total trials completed, strategy score, emotional/ social cognition: number of correctly identified emotions, accuracy per emotion (e.g., happiness, fear),memory, total correct responses, trials to success/trials to criterion, mean correct latency,% correct, delayed recall accuracy (e.g., reaction time).	4–90.	Internal consistency, construct validity [58], discriminant validity [59]	Developed as digital
**Tower of London Test** [60]	measure planning and problem-solving abilities	Solve a problem using two wooden towers and diameter balls by reaching the examiner’s tower abstraction within a specified time and number of moves. Different difficulty levels and versions (different numbers of balls) exist.	children with neurodevelopmental disorders	Total score, planning time, task level achieved, execution time	6–80 (digital version intended for 5–53)	Cronbach’s alpha convergent validity, discriminant validity [61]	Transferred to digital format
**Wisconsin Card Sorting Test for children (WCST)** [62]	assess abstract reasoning, cognitive flexibility, and EFs by evaluating the ability to adapt to changing sorting rules	Four stimulus cards and 128 response cards with printed objects that differ in number, color, and shape: The child matches the response cards to the stimulus cards with correct or incorrect feedback. A short form with 64 cards is available.	adults and adapted for children and adolescents	Total number of correct answers, total errors, perseverative responses, perseverative errors, non-perseverative errors, categories completed, number of trials to complete the category, % conceptual level response, failure to maintain set.	6.5–89.0	Norms are available for children 6 months–6 years [62]	Transferred to digital format
**The Birthday Task Assessment** [63]	assess performance in a complex, multistep task requiring EF abilities	A role-playing scenario related to a birthday party. The child must complete three tasks of varying difficulty according to specific rules: prepare two sandwiches with peanut butter and jelly, wrap two birthday presents, and prepare a card for the birthday party.	children with neurodevelopmental disorders.	Total time, broken rules, errors (omission, object substitution, action addition, total errors)	8–16	Interrater reliability [63]	No digital version available
**Adaptive Cognitive Evaluation Explorer (ACE-X)** [64]	assess cognitive abilities, including working memory, attention, and cognitive flexibility. The assessment was adapted from the Adaptive Cognitive Evaluation-Classroom (ACE-C).	This mobile EF assessment tool includes 15 tasks in real-world settings. An incorporated algorithm enables repeatedly administering the same tasks without losing sensitivity to low performance levels.	anyone 7–107 years experiencing cognitive difficulties	Processing speed, working memory, inhibitory control, and cognitive flexibility	7–107	Intraclass correlation coefficients, test–retest reliability, concurrent validity [64]	Developed as digital

### 3.2. Ecological Validity of Traditional Tools Versus Digital Platform Assessments

**Figure 2:**
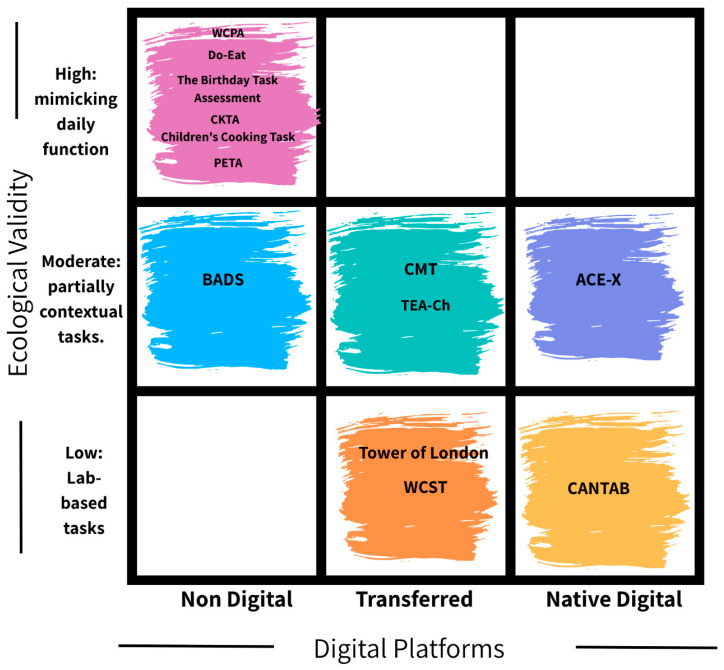
Conceptual map of functional cognitive assessments.

The digital transformation of assessments necessitates an evaluation of their ecological validity and digital platforms. Figure 2 presents a conceptual map illustrating the positioning of functional cognitive assessments for children and adolescents along two key dimensions: ecological validity and digital platforms. The vertical axis reflects how well each tool simulates real-life tasks, ranging from low (lab-based tasks) to high (real-life or role-play scenarios mimicking daily functioning). The horizontal axis classifies tools by their digital platforms: non-digital (paper-based only), transferred to digital (originally analog, now also in digital format), or developed as native digital platforms. Assessments such as the WCPA, Do-Eat, and Birthday Task Assessment are in the top-left quadrant, indicating high ecological validity with no digital availability. Assessments like the CMT and Test of Everyday Attention for Children (TEA-Ch) are in the moderate-middle quadrant, suggesting partial contextual relevance and availability in transferred digital formats. Only the ACE-X is in the top-right quadrant, representing both high ecological validity and a native digital format. Conversely, lab-based tools, such as the Tower of London, WCST, and CANTAB, occupy the lower cells, reflecting lower contextual relevance despite varying digital availability.

### 3.3. Scoring Metrics Across Cognitive Domains

We conducted a classification process based on content analysis of the assessment characteristics to better understand the distribution of scoring metrics used across assessment tools for functional cognition in children and youth. We reviewed and categorized each assessment according to the type of cognitive functions it evaluated, with specific attention to whether performance-based scoring metrics were used to quantify ability. Guided by widely accepted theoretical frameworks in neuropsychology and rehabilitation sciences, we identified eight core cognitive domains for grouping the assessments. These domains reflect the primary constructions evaluated by most tools in the reviewed table and are consistent with contemporary EF and cognitive performance models.

#### 3.3.1. Rationale for the Eight-Domain Classification

The eight-domain classification was designed to reflect the multifaceted nature of functional cognition, recognizing that successful performance of daily activities requires coordinated use of multiple cognitive abilities. By grouping assessment tools according to their primary focus, such as EFs, attention, memory, or sequencing and organization, this framework clarifies which abilities an assessment directly measures and which measures only infers indirectly through performance patterns, strategy use, or errors.

#### 3.3.2. The Seven Domains

- Executive functions: This broad category encompasses planning, inhibition, self-monitoring, cognitive flexibility, and strategy use. Most instruments reviewed assessed at least one executive component, justifying the domain’s centrality.
- Attention: Sustained, selective, and divided attention were clustered as a distinct domain, given that several assessments exclusively targeted attentional capacity independent of broader executive processes.
- Processing speed: Assessments measuring reaction time, information processing efficiency, and cognitive fluency were grouped under this domain.
- Problem-solving and planning: This domain included assessments that required multistep reasoning, hypothesis generation, and goal-directed behavior (e.g., Tower of London, WCPA).
- Sequencing and organization: Specific to everyday tasks requiring ordered steps and spatial-temporal organization, this domain emerged from assessments such as the Do-Eat and cooking tasks.
- Memory: This domain encompasses immediate recall and the ability to hold and manipulate information during task performance. It included encoding, storage, and information retrieval processes essential for everyday functioning. Assessment tools that capture this domain include the CMT, ACE-X, and selected CANTAB subtests.
- Emotional/social cognition: Although assessed less frequently than the other domains, we retained this domain to reflect assessments that include affect recognition and social reasoning, particularly in computerized batteries like the CANTAB.

**Figure 3:**
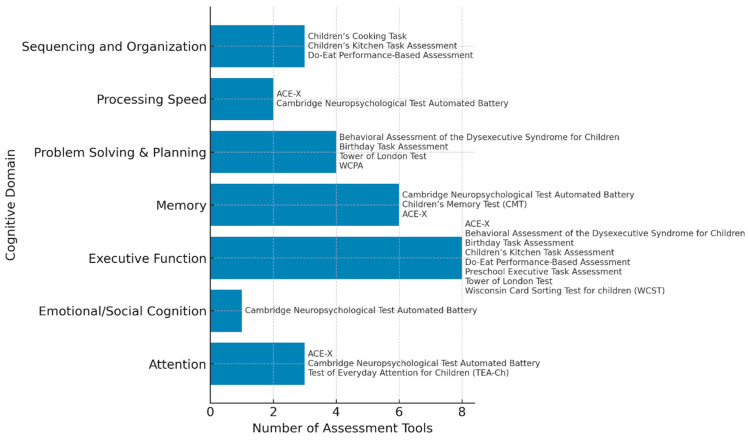
Frequency of assessment tools using scoring metrics across eight cognitive domains.

We mapped each assessment tool to one or more identified domains, depending on the tool’s primary constructs and subtests. Assessments appearing in multiple domains were counted in each relevant category. To quantify the frequency of scoring metrics used across domains, we calculated the number of distinct assessments that utilized scoring metrics within each domain. A bar chart was then constructed (Figure 3) to represent this distribution visually. Each bar in the chart corresponds to a cognitive domain; the bar length indicates the number of assessments using scoring metrics in this domain. The names of the assessment tools contributing to each domain are displayed alongside the bars for clarity and transparency.

## 4. Discussion

This conceptual review aimed to evaluate the transformation of traditional functional cognitive assessments into digital platforms. The data extraction yielded three primary units of analysis: digital transformation trends, ecological validity and digital availability, and scoring metrics across cognitive domains.

### 4.1. Digital Transformation Trends

This study examined 13 functional cognition assessments in the context of daily life. These assessments, designed for children and adolescents with various conditions (e.g., neurodevelopmental disorders), cover a wide age range. They have been standardized and validated for reliability and validity. Interestingly, very few are digitally available, and many were not originally developed for digital formats. This lower digital availability likely reflects the difficulty of capturing daily settings digitally and its inadequacy for evaluating certain cognitive abilities. Assessments such as the CCT [32] and the Do-Eat [49] assess cognitive performance in complex daily activities using daily scenarios, a feature that is difficult to replicate on a digital platform. Likewise, the WCPA [31,37] relies on direct observation of performance and strategy use, two crucial elements that could be compromised if the assessment were digitized.

Specifically, *cognitive strategies*—mental action plans individuals use to approach challenging tasks systematically—are considered integral to typical learning and performance. These strategies can often be observed during or immediately after the execution of a task and are crucial for children and adolescents to acquire motor skills and achieve occupational performance [65,66]. Their need for a therapist’s direct observation makes transforming these traditional assessments into digital platforms particularly challenging.

Conversely, technological platforms offer inherent advantages, such as enhanced data precision and efficiency. They can capture fine-grained performance metrics (e.g., reaction times, eye-tracking) by recording micro-level behavioral data [67]. Furthermore, digital platforms can reduce therapist workload and enable large-scale data collection. As Condy et al. [68] demonstrated, tablet-based assessments allow efficient testing with reduced therapist involvement.

However, according to previous studies, therapists may not be comfortable moving away from traditional methods due to concerns about accuracy and personal interaction [69,70]. Considering the potential of technological platforms, assessments that are indeed digitally available could be incorporated into clinical practice.

### 4.2. Ecological Validity and Digital Availability

Positioning the assessments along two axes—one examining ecological validity, and the other digital examining availability—allows analysis of their practical utility. Our analysis revealed a key challenge: Assessments that effectively represent real-world daily performance through engaging, ecological scenarios are often the most difficult to replicate in a digital environment without compromising validity.

Our findings illustrate this digital trend, showing that assessments with high ecological validity (e.g., WCPA, Do-Eat, Birthday Task) tend to lack digital availability. The ACE-X was an exception, demonstrating ecological validity and native digital format. Other assessments, including the CMT and TEA-Ch, fell in the middle; they showed partial contextual relevance and availability in transferred digital formats. The Tower of London, WCST, and CANTAB had lower contextual relevance despite their varying degrees of digital availability.

Innovative technological advancements, particularly in VR, have opened new avenues for enhancing the ecological validity of cognitive assessments [71]. Current VR environments show promise for improving ecological validity because they offer more realistic evaluations of daily cognitive abilities. The VR-based tasks have demonstrated correlations with traditional EF tests but better reflect everyday behavioral functioning [15]. Therefore, incorporating more advanced technology into such assessments may increase the ability to transform them into digital platforms.

### 4.3. Scoring Metrics Across Cognitive Domains

The scoring metrics classification process resulted in seven core cognitive domains: EFs, attention, processing speed, problem-solving and planning, sequencing and organization, memory, and emotional/social cognition. These classifications clarify the specific cognitive constructs each assessment addresses. They also highlight which abilities are directly observable during task performance and which are inferred indirectly from performance patterns, strategy use, or errors.

Recognizing the variability across these domains underscores the multifaceted nature of functional cognition and the diverse skill set children and adolescents need to navigate daily life successfully [5,72]. Within the context of this review, the eight-domain framework serves as a comparative tool for mapping assessments, identifying coverage gaps, and guiding the selection or development of tools—particularly in the transition from traditional to digital platforms—to ground clinical and research decisions in a comprehensive understanding of cognitive demands.

### 4.4. Limitations and Future Directions

There are several limitations to this study. First, the review identified only 13 studies. Although this small number likely reflects the general scarcity of digitally available ecological assessments, future research could include, for instance, gray literature, such as doctoral dissertations and conference proceedings, which may report on emerging digital tools before they appear in peer-reviewed publications. Second, the complex definition of *functional cognition* complicates the application of inclusion criteria. Because the term is relatively new [3,4,73], we included many studies based on our interpretation that they measured functional cognition—even if they did not explicitly use the term. It would be useful if future studies developed criteria for measuring functional cognition. Finally, future studies should establish the validity and reliability of these digital tools by examining their measurement invariance, usability, and social validity with children and caregivers in naturalistic settings, which could contribute to the future development of digital functional cognitive assessments.

## 5. Conclusions

Although research has explored the digitalization of traditional assessments, the specific transformation of performance-based functional cognition assessments into digital tools remains a gap in the literature. Transforming functional cognitive assessments into digital formats represents a significant opportunity to enhance their accessibility, accuracy, and inclusivity [74,75]. However, to justify investment in their development and implementation, technology-based tools should not only replicate but also enhance traditional assessment features [28].

The conceptual model developed in this review is grounded in three core evaluative dimensions: technological availability, ecological validity, and scoring metrics across cognitive domains. By integrating these dimensions, our model offers a structured framework for mapping and comparing functional cognitive assessments for children and adolescents. Positioning tools along the axes of ecological validity and digital availability and overlaying their distribution of scoring metrics allows clearer differentiation between assessments that may appear similar in purpose but diverge in their capacity to capture real-world cognitive performance or to leverage technological platforms.

This perspective facilitates identifying gaps, such as the absence of highly ecologically valid tools in native digital formats. It provides a basis for examining how well existing assessments align with contemporary clinical, educational, and technological demands.

Building on this framework, the model sharpens our ability to distinguish between specific assessments, highlighting their relative strengths, limitations, and potential for digital transformation. The findings derived from this framework have direct implications for practice and future development. For therapists, this model can guide the selection of the most appropriate assessments. For technology developers, it can inform the incorporation of more advanced tools capable of capturing nuanced behavioral observations in realistic daily contexts. For certain tools, such as the WCPA, it may be possible to incorporate digital features through a data-logging platform that records responses on a touchscreen or captures user actions via video, thereby supporting more efficient scoring and reducing the cognitive load of the examiner.

Finally, for researchers, the model encourages the continued exploration of traditional assessments’ ecological validity while recognizing the unique research advantages of computerized tools, particularly VR-based platforms that automatically record process-oriented data. Such tools save valuable interaction time with participants while providing detailed step-by-step insights into functional cognition and its underlying cognitive components, most notably EFs, thereby enhancing the efficiency and depth of cognitive evaluation.

## Data Availability

The original contributions presented in this study are included in the article/Supplementary Materials. Further inquiries can be directed to the corresponding author.

## Supplementary Materials

The following supporting information can be downloaded at: https://www.mdpi.com/article/10.3390/children12101384/s1, TITAN Guideline Checklist. Reference [76] is cited in Supplementary Materials.

## Abbreviations

The following abbreviations are used in this manuscript:

ADL	Activity of daily living
ACE-X	Adaptive Cognitive Evaluation Explorer
BADS	Behavioral Assessment of the Dysexecutive Syndrome
CANTAB	Cambridge Neuropsychological Test Automated Battery
CCT	Children’s Cooking Task
CMT	Children’s Memory Test
EFs	Executive functions
TEA-Ch	Test of Everyday Attention for Children
VR	Virtual reality
WCPA	Weekly Calendar Planning Activity
WCST	Wisconsin Card Sorting Test
