# Supplementary material for: Can Functional Cognitive Assessments for Children/Adolescents Be Transformed into Digital Platforms? A Conceptual Review
Source: Children (Basel). 2025 Oct 14;12(10):1384. doi: 10.3390/children12101384 (PMC12564286; doi:10.3390/children12101384)
Supplement: Supplementary file 1 [file children-12-01384-s001.zip › children-3868207-supplementary.pdf]

Can Functional Cognitive Assessments for children/adolescents be transformed into Digital Platforms? A Conceptual Review

TITAN Guideline Checklist[1]

Topic	Item	Description	Page Number
Artificial Intelligence (AI) (some journals may prefer this in the methods and/or acknowledgements section, and it should also be declared in the cover letter)	Declaration of whether any AI was used in research and manuscript development	Yes	5
	1a Purpose and Scope of AI Use	Elicit Pro was used to identify and analyze literature related to the digital transformation of performance-based cognitive assessments. The purpose was to support a systematic conceptual review by screening and synthesizing recent evidence.	5-6
	1b AI Tool(s) and Configuration	The tool employed was Elicit Pro (AI-powered research assistant, Pro license). It searched across more than 126 million open-access and non-open-access academic papers. Filters were set for the last 10 years and English-language publications.	5-6
	1c Data Inputs and Safeguards	Structured search queries were entered into Elicit's 'Find Papers' feature. The queries focused on the transformation of traditional functional cognitive assessments into digital platforms. Output included titles, abstracts, citations, and metadata for screening (e.g. Reasoning and Supporting quotes for "ecological validity" ;"Remote administration"; "Transformation"; "Performance based"; Name of	5-6

		the assessment tool; and DOI link)	
	1d Human Oversight and Verification	All data collection, screening and evaluation were performed by the research team. Abstracts and full texts were screened, and inclusion/exclusion criteria were applied manually to mitigate over- or under-retrieval by the AI system. The team reviewed every AI output and checked every DOI link, fact and clinical accuracy.<br>AI-generated figures were manually and carefully edited.	66
	1e Bias, Ethics and Regulatory Compliance	Potential algorithmic bias was addressed by applying human verification and exclusion criteria. Studies unrelated to assessments, or involving adult-only populations, were excluded. This process ensured methodological rigor and compliance with ethical research standards.	6
	1f Reproducibility and Transparency	The search and screening process was documented step by step. From 240 initial items, 45 were retained after abstract screening, and 13 after full-text review. Each tool was analyzed once, and extraction tables were created for transparency and replicability. Although the specific search prompts entered to Elicit are not publicly available, the inclusion/exclusion criteria and extraction tables are fully documented to support transparency and partial reproducibility.	6

Can Functional Cognitive Assessments for children/adolescents be transformed into Digital Platforms? A Conceptual Review

[1]Agha, R.A.; Mathew, G.; Rashid, R.; Kerwan, A.; Al-Jabir, A.; Sohrabi, C.; Franchi, T.; Nicola, M.; Agha, M.; the TITAN Group. Transparency in the Reporting of Artificial Intelligence – The TITAN Guideline. Premier Journal of Science 2025, 10, 100082. https://doi.org/10.70389/PJS.100082.
